# Maximizing acceptance of clinical pharmacy recommendations to reduce length of hospital stay in a private hospital from Amman, Jordan

**DOI:** 10.1186/s12913-021-06966-4

**Published:** 2021-09-08

**Authors:** Loai M. Saadah, Amer H. Khan, Syed Azhar Syed Sulaiman, Iman A. Bashiti

**Affiliations:** 1 Discipline of Clinical Pharmacy, School of Pharmaceutical Sciences, Universiti Sains Malaysia, 11800 Gelugor, Penang Malaysia; 2 Department of Clinical Pharmacy, Ibn Al Haytham Hospital, Amman, Hashemite Kingdom of Jordan; 3 Department of Clinical Pharmacy, Faculty of Pharmacy, Applied Sciences University Pharmacy, 11931 Amman, Hashemite Kingdom of Jordan

**Keywords:** Pharmacists, Clinical pharmacy interventions, Acceptance, Length of stay, Neural networks, Jordan, Patient readmission, Hospitalization, Private hospital

## Abstract

**Background:**

Clinical pharmacy interventions (CPI) usually require prior medical authorization. Physicians approve 80% of CPI and reject 20%. If pharmacists show that physicians should authorize all 100% CPI, the profession will step closer to a fully independent prescriber status. This study used an artificial neural network (ANN) model to determine whether clinical pharmacy (CP) may improve outcomes associated with rejected CPI.

**Method:**

This is a non-interventional, retrospective analysis of documented CPI in a 100-bed, acute-care private hospital in Amman, Jordan. Study consisted of 542 patients, 574 admissions, and 1694 CPI. Team collected demographic and clinical data using a standardized tool. Input consisted of 54 variables with some taking merely repetitive values for each CPI in each patient whereas others varying with every CPI. Therefore, CPI was consolidated to one rejected and/or one accepted per patient per admission. Groups of accepted and rejected CPI were compared in terms of matched and unmatched variables. ANN were, subsequently, trained and internally as well as cross validated for outcomes of interest. Outcomes were length of hospital and intensive care stay after the index CPI (LOSTA & LOSICUA, respectively), readmissions, mortality, and cost of hospitalization. Best models were finally used to compare the two scenarios of approving 80% versus 100% of CPI. Variable impacts (VI) automatically generated by the ANN were compared to evaluate the effect of rejecting CPI. Main outcome measure was Lengths of hospital stay after the index CPI (LOSTA).

**Results:**

ANN configurations converged within 18 s and 300 trials. All models showed a significant reduction in LOSTA with 100% versus 80% accepted CPI of about 0.4 days (2.6 ± 3.4, median (range) of 2 (0–28) versus 3.0 ± 3.8, 2 (0–30), *P*-value = 0.022). Average savings with acceptance of those rejected CPI was 55 JD (~ 78 US dollars) and could help hire about 1.3 extra clinical pharmacist full-time equivalents.

**Conclusions:**

Maximizing acceptance of CPI reduced the length of hospital stay in this model. Practicing Clinical Pharmacists may qualify for further privileges including promotion to a fully independent prescriber status.

## Background

Clinical pharmacist interventions (CPI) reduced length of stay in various settings [1, 2]. Additionally, they shortened intensive care and hospital resource utilization [3]. Researchers further showed a significant improvement in survival with CPI [4, 5]. Moreover, they have enormous financial benefits to payers [6–9]. In contrast, CPI had conflicting effects on reducing readmissions, as some did reduce readmissions while others failed to do the same [10–13]. Impact on the duration of hospitalization seemed negligible in a Scandinavian emergency department as well [14]. Therefore, the effect of CPI may vary among institutions.

Now, CPI usually requires prior authorization of a physician and hence is associated with acceptance rates in the range of 80% [15, 16]. Several factors garner higher acceptance including class of medications and CPI, diseases, and specialties of physicians, as well as experiential background of clinical pharmacists [16, 17]. For example, in our previous study in a governmental medical military setting, higher acceptance correlated positively with more than 3 years pharmacy experience, board certification, and a Doctor of Pharmacy degree. Critical care, pediatrics and oncology recommendations were more accepted as well. Moreover, recommendations to stop medications were significantly more likely to be authorized. Finally, CPI were more likely successful if they were about antibiotics [16]. Similarly, Mann et al. showed that stopping a medication is more likely to be successful in the context of managed care for members of a Medicare Advantage Plan [17]. Additionally, they found that there was institutional regional variation in the acceptance of clinical pharmacy recommendations. The same group also found that heart failure recommendations were more likely be accepted than diabetes CPI [17]. On the other hand, despite resistance to pharmacist prescribing, more evidence is building up as to its value for patient care [18, 19]. Therefore, although we understand that prescribing is a complex process with multiple factors playing a role, it is of primary importance to reconcile the 20% disagreement on pharmacotherapy that exists between pharmacists and physicians. This should be undertaken in any effort to broaden the scope of pharmacy prescribing to fully independent status. Up to this point, scarcely, if at all, does the literature evaluate this 20% discordance due to the many variables (can appreciate from the 54 variables in this study) involved and extreme difficulty (to show the difficulty imagine you want to approve a CPI anyway when a physician reject it!) in conducting such an experiment.

Artificial neural networks (ANN) are a very powerful tool that we use to resolve complex doubts by analyzing real usual care data [20]. These models evade the need to rigorously intervene and, hence, make sophisticated protocols of blinding and randomization of patients often unnecessary [20].

In this study, we used ANN to evaluate the outcomes associated with maximizing the acceptance of CPI from 80% to almost 100% and hence whether pharmacists qualify for a full independent prescribing authority. Specifically, would accepting more CPI potentially improve healthcare outcomes such as length of hospital and intensive care stay, reduce readmissions, improve survival, or reduce costs? We specifically have the following outcomes to assess: Primary Outcome was Length of hospital stays after the index CPI (LOSTA) as a continuous variable. Secondary objectives included lengths of Intensive care unit (ICU) stay after the index intervention (LOSICUA) and cost of hospitalization (both continuous) as well as readmissions and mortality (both categorical).

## Methods

### Study design

It was a usual acute care, eight-month-long, two-phase, non-interventional retrospective study**.**

### Study institution and setting

This was a 100-bed, tertiary- and acute-care private hospital in Amman, Jordan.

### Clinical pharmacy team

Our clinical pharmacy team consisted of three clinical pharmacists with 4, 7 and 8 years of clinical pharmacy experience. All are doctor of pharmacy or Master of Clinical Pharmacy certificate holders. Clinical pharmacist with the shortest experience was also a recent hire. These clinical pharmacists cover about 50% of admissions. Remaining 50% are those in weekends, admitted and discharged overnight, or from uncovered units such as obstetrics and gynecology. Clinical pharmacists rotated every 2 months on the remaining units which are medical, surgical, intensive care unit and pediatrics. All medical specialties were represented in these departments except for psychiatry and oncology. Clinical pharmacists made their proactive rounds daily during which most recommendations (84%) were made. Only 16% of their recommendations were consult-based.

### Study panel

This was made up of 4 academics from 2 universities one local and one international**.** These professors are separate from the clinical pharmacy team, and they were presented with blind full information of the individual cases in a detailed SOAP format. Based on these notes they independently either agreed or disagreed with the clinical pharmacist recommendation. So basically, the academic does not know which was the decision of the physician and which was the recommendation of the clinical pharmacist.

#### Study subjects: inclusion and exclusion criteria

Consecutive patients of all ages were included when admitted to the study institution with CPI to outpatients excluded due to the unavailability of outcomes.

#### Data collection

Team documented daily usual recommendations using a standardized Microsoft Excel 2010 tool (version 14.0.4374.1000). This tool collected all variables for each CPI using clear definitions as we explain in the definitions section. Variables documented for each CPI can be found in Table 1 (matched between accepted and rejected CPI) and 2 (unmatched). Each patient varied; some stayed for a day, and some stayed longer, CPI were collected during that patient stay. Length of stays were given in hours by IT for each patient for the respective admission, 1 month follow up was done to see if the patients were readmitted. Costs were also real costs incurred to patients or their insurance during the admission. Status of whether alive, improved, or dead was given by IT. In the study database each row constituted a CPI. For each patient, there were multiple CPI in each admission and patients may have been admitted more than once during the study period. Hence, for each patient during a given admission, some variables value may have been merely a repetition with every CPI allowing us to use that value in a consolidated CPI. Other variables varied with every CPI. Therefore, we consolidated the data to a maximum of one accepted and one rejected per patient per admission using arbitrary ordinal scores for the variables changing with every CPI. These scores were calculated with the details provided in the definition’s subsection. Medication information from the local pharmacy and stock management databases were confirmed. Similarly, the laboratory information from the local laboratory database was reported. Lengths of hospitalization, readmissions, and costs were collected from the local admissions database. Lengths of hospitalization and ICU stay were known in days from the IT system for each patient. Readmissions were assessed and measured in the 30 days following discharge. Costs of admission incurred to patient, or their insurance were that real paid by each during that admission. All IT databases were built by Microsoft Access 2000 (version 9.0.6926 SP-3) and managed by the information technology (IT) department in the hospital. Status of the patient upon discharge, whether alive or deceased, was provided by the IT department.

**Table 1:** Matched Variable for the 684 Consolidated Recommendations

Variable	Accepted (*N* = 519)	Rejected (*N* = 165)	***P***-value
**Demographic Factors**			
Patient Age in Years	44 (0.05–92)	53 (0.05–92)	
Gender	M: 282 (54%)	M: 96 (58%)	
Nationality	Jordan: 477 (92%)	Jordan: 150 (91%)	
Age Group	Adult: 381 (73%)	Adult: 127 (77%)	
Criticality	Critical: 151 (29%)	Critical: 55 (33%)	
Elderly (> 84 years old)	Yes: 29 (6%)	Yes: 10 (6%)	
Insurance status	Insured: 351 (68%)	Insured: 108 (66%)	
Hospital Stay, Before Index	1.0 (0.0–38.0)	1.0 (0.0–10.0)	
ICU^a^ Stay, Before Index	0.0 (0.0–38.0)	0.0 (0.0–9.0)	
**Risk of Hospitalization Factors**			
Anticholinergic	Yes: 1 (< 1%)	Yes: 1 (< 1%)	
Antiarrhythmic	Yes: 7 (1%)	Yes: 3 (2%)	
Dementia	Yes: 32 (6%)	Yes: 15 (9%)	
Anemia	Yes: 22 (4%)	Yes: 13 (8%)	0.065
Heart Failure	Yes: 14 (3%)	Yes: 9 (6%)	0.087
Two Anti-hypertensives	Yes: 37 (7%)	Yes: 19 (12%)	0.073
Three Anti-hypertensives	Yes: 13 (3%)	Yes: 9 (5%)	0.061
Beta Blocker	Yes: 55 (11%)	Yes: 20 (12%)	
Benzodiazepines	Yes: 18 (4%)	Yes: 9 (6%)	
Tricyclic Antidepressants	Yes: 0 (0%)	Yes: 0 (0%)	
Non-Green Antibiotics	Yes: 242 (47%)	Yes: 87 (53%)	
Surgical case	Yes: 56 (11%)	Yes: 18 (11%)	
**Clinical Pharmacy Recommendation Types**			
Non-drug Recommendation	Yes: 198 (38%)	Yes: 77 (47%)	0.052
Change in Drug Form	Yes: 70 (14%)	Yes: 30 (18%)	
Change in Dose	Yes: 376 (72%)	Yes: 123 (75%)	
Change in Frequency	Yes: 297 (57%)	Yes: 98 (59%)	
Change in Route	Yes: 129 (25%)	Yes: 45 (27%)	
Change in Duration	Yes: 71 (14%)	Yes: 17 (10%)	
Safety Related	Yes: 161 (31%)	Yes: 56 (34%)	
Miscellaneous	Yes: 287 (55%)	Yes: 98 (59%)	
Not Determined	Yes: 34 (7%)	Yes: 9 (5%)	
**Scores**			
Problem Complexity	1.0 (0.0–1.0)	0.9 (0.0–1.0)	
Problem Intention	1.0 (0.0–1.0)	1.0 (0.5–1.0)	
Clinical Domain	1.0 (0.0–1.0)	1.0 (0.7–1.0)	
Prescribing Step	1.0 (0.0–1.0)	0.4 (0.0–1.0)	
Outcomes Driven	0.2 (0.0–1.0)	0.3 (0.0–1.0)	
Diagnosis Revised	0.0 (0.0–1.0)	0.0 (0.0–1.0)	

^a^*ICU* Intensive Care Unit

#### Definitions

Phase I, from January 1st to March 31st, 2019, was a pilot to estimate the needed sample size. Phase II, was conducted from April 1st to August 31st, 2019. The study began January 1st, 2019 but enrollment of every case was different. For each patient, the length of the study differed as we have collected the information for that patient during his stay at our hospital and for 1 month post discharge. So, this makes the last day we collected patient post discharge or readmission data September 30th, 2019. In other words, we had 1 month follow up to catch any new readmissions for that patient. Again, Table 1 (36 matched variables) and Table 2 (18 unmatched) summarize all the 54 variables included in this model. As the reader may appreciate, some of these were continuous like age in years, total number of CPI, or the length of hospital or intensive care stay before the first index CPI in days (LOSBI and LOSICUBI). However, the majority were categorical. For example, in Table 1, our reader can see that some CPI was made in critical cases in the intensive care unit whereas others made in non-critical medical cases. Most categorical variables are listed such that a given factor is either present (Yes) or absent (No). For example, there were 55 CPI (11%) in the accepted group and 20 CPI (12%) in the rejected group on beta blocker medications. Obviously, beta blockers were matched between accepted and rejected CPI groups. In terms of the medicines and their types we used the current American Society for Health System Pharmacists (AHFS Drug Information) taxonomies from Lexicomp (Lexi-drugs online [database on the Internet]. Hudson (OH): Lexicomp, Inc.; 2020; cited 26 Aug 2020]. Available from: http://online.lexi.com. Subscription required to view). Polypharmacy was a dichotomous variable with 1 for ≥8 regular medications and 0 otherwise. This was a study-panel, agreed-upon definition of polypharmacy. Previously published literature was used to identify medication and disease-related variables predicting hospitalization due to drug adverse effects [21].

**Table 2:** Summary Statistics of Un-Matched Variables and Outcomes for the 684 Consolidated Recommendations

Variable	Accepted (*N* = 519)	Rejected (*N* = 165)	***P***-value
**Demographic Factors**			
Phase of CPI^a^	Phase II: 271 (52%)	Phase II: 64 (39%)	0.003
Total Number of CPI	2 (1–38)	3 (1–38)	< 0.001
**Risk of Hospitalization Factors**			
Poly-pharmacy	Yes: 80 (15%)	Yes: 40 (24%)	0.009
Multi-comorbidities	Yes: 131 (25%)	Yes: 59 (36%)	0.009
Vascular Disease	Yes: 39 (8%)	Yes: 22 (13%)	0.022
ACEI/ARB	Yes: 48 (9%)	Yes: 25 (15%)	0.032
Diuretic	Yes: 42 (8%)	Yes: 22 (13%)	0.044
Renal Disease	Yes: 90 (17%)	Yes: 44 (27%)	0.009
Liver Disease	Yes: 14 (3%)	Yes: 11 (7%)	0.018
High Alert Medication	Yes: 189 (36%)	Yes: 76 (46%)	0.027
**Clinical Pharmacy Recommendation Types**			
Drug Prescribing	Yes: 326 (63%)	Yes: 129 (78%)	< 0.001
Efficacy Related	Yes: 180 (35%)	Yes: 83 (50%)	< 0.001
Stop a Medication	Yes: 137 (26%)	Yes: 69 (42%)	< 0.001
Add a Medication	Yes: 108 (21%)	Yes: 48 (29%)	0.027
**Scores**			
Consultancy	0.0 (0.0–1.0)	0.0 (0.0–1.0)	0.029
Rejection	0.0 (0.0–0.8)	0.5 (0.0–1.0)	< 0.001
Combined CP Success	0.9 (0.6–1.0)	0.9 (0.6–1.0)	< 0.001
Physician Rejection	0.2 (0.0–0.8)	0.2 (0.0–1.0)	< 0.001
**Outcomes**			
Hospital Stay, Total	3.0 (0.5–59.0)	4.0 (0.5–32.0)	0.066
ICU Stay, Total	0.0 (0.0–59.0)	0.0 (0.0–17.0)	0.412
Hospital Stay, After Index	1.0 (0.0–43.0)	2.0 (0.0–30.0)	0.232
ICU^a^ Stay, After Index	0.0 (0.0–43.0)	0.0 (0.0–16.0)	0.119
Readmission	Yes: 80 (15%)	Yes: 27 (16%)	0.770
Mortality	Died: 8 (2%)	Died: 5 (3%)	0.222
Cost of Hospitalization (JD^a^)	1149 (94–23,686)	1388 (200–22,823)	0.005

^**a**^*CPI* Clinical Pharmacy Intervention, *ICU* Intensive Care Unit, *JD* Jordanian Dinar (equals to 1.41 US Dollars)

Non-green antibiotics are a special traffic signal classification we use in antimicrobial stewardship programs. For example, meropenem is red, cefepime is orange, and levofloxacin is green. Red antibiotics are the most serious and require rigorous stewardship while green antibiotics undergo only limited control.

After a quick revision of all types of recommendations made, it was found that there were significant differences between safety and efficacy CPI, i.e., those made to prevent an adverse effect and those made to improve the efficacy of a treatment.

Another area of difference was between orders made to add or stop a medication. Differences were absent or little for the other types, and therefore, these were grouped under miscellaneous. Unreported or difficult to categorize CPI were grouped under “Not determined”.

Each non-consolidated individual CPI would be made to the physician and the physicians in whole would accept 80% and reject 20%. Patients normally would be counselled on these changes and in our setting normally would agree to all final decisions made by the physician. Scores were calculated to consolidate all CPI to a maximum of 1 accepted and/or rejected per patient per admission. These scores took a value between 0 and 1. For example, CPI was considered complex (took the value of ‘1’) if it involved putting two or more pieces of information together. Simple CPI (took a value of ‘0’) if based on one direct piece of information; for example, if the patient was hypotensive, the simple CPI was to stop the antihypertensive drug. Follow-up CPI (took a value of ‘0.5’) would be based on a check of a new investigation and hence follow-up on a previous CPI. Therefore, problem complexity score was a total average % of all; complex, simple, and follow-up CPI made for a given patient during an admission. As the reader may see, the two groups were comparable in the complexity of CPI (1 versus 0.9 for accepted and rejected CPI groups, respectively, and ranges in brackets). Similarly, Problem intention score was a total % average of errors and problems documented for a given patient during the admission. CPI was a problem (took a value of ‘1’) if the physician tried to defend their original plan. It was an error (took a value of ‘0’) if the physician immediately agreed or explicitly clarified that they made a mistake. Clinical domain score was a % average of clinical (value of 1) versus operational (value of 0) CPI for that admission. An example of a clinical CPI is changing a dose. Whereas an example of operational CPI is to reuse a given stable intravenous medication vial for multiple doses. Clinical Prescribing step score was a % average of CPI for that admission made to the prescribing step in the medication use process. The consultancy score was a % average of the clinician approaching the clinical pharmacist (value of ‘1’) versus the clinical pharmacist approached the clinician (value of ‘0’) during that admission. Outcomes driven score was a % average of CPI made based on outcomes versus those made with guidelines for that admission. Rejection score was % of CPI rejected in that admission. Combined clinical pharmacy (CP) success score is the % average of clinical pharmacist’s successful CPI averaged over that admission. The physician rejection score was the % of physicians’ rejection rates during the whole study period averaged for that admission recommendations. The diagnosis revision score was the % of all diagnoses that were revised or changed by the end of the admission. The acceptance or rejection of the CPI was entered as a dichotomous input variable.

Finally, outcomes studied were LOSTA, length of stay in the intensive care unit (LOSICU), LOSICUA, readmission, mortality, and cost of admission.

#### Data analysis

**Fig. 1:**
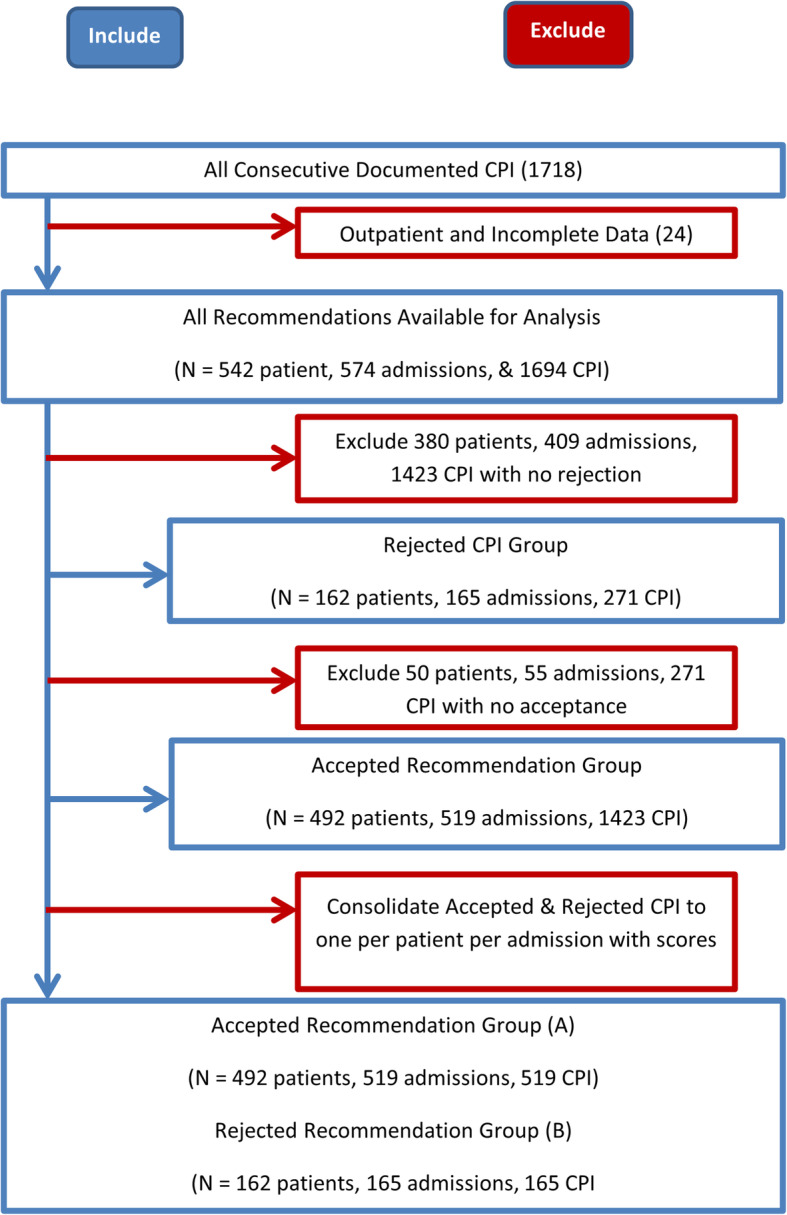
Summary of Inclusion of All Consecutive Patients with Documented Recommendation until final consolidation into one accepted and/or one rejected recommendation per patient per admission

Analysts consolidated the data for a total of one CPI in accepted or rejected groups per admission per patient. Variables for different CPI were simply set to the matching values or calculated scores described in the definitions section. Final inclusion and refinement of scenarios are presented in Fig. 1. Our reader can see that a total of 1694 CPI was finally consolidated to 684 CPI, 519 in the accepted and 165 in the rejected groups, respectively. Univariate analyses were conducted to compare the rejected and accepted CPI groups (Tables 1 and 2). Finally, the research team built, trained, tested, and cross validated ANN for the main and various outcomes.

#### Statistical analysis

The data was analyzed using Stat Tool, version 6.3.0 (Palisades Corp) to generate *P*-values using Mann Whitney U Test unpaired groups for continuous data, Chi-squared (χ^2^) test for categorical variables, McNemar Test for categorical outcomes during re-assignment ANN analyses, Wilcoxon Mann Whitney Test for continuous paired outcomes during re-assignment.

#### ANN model

**Fig. 2:**
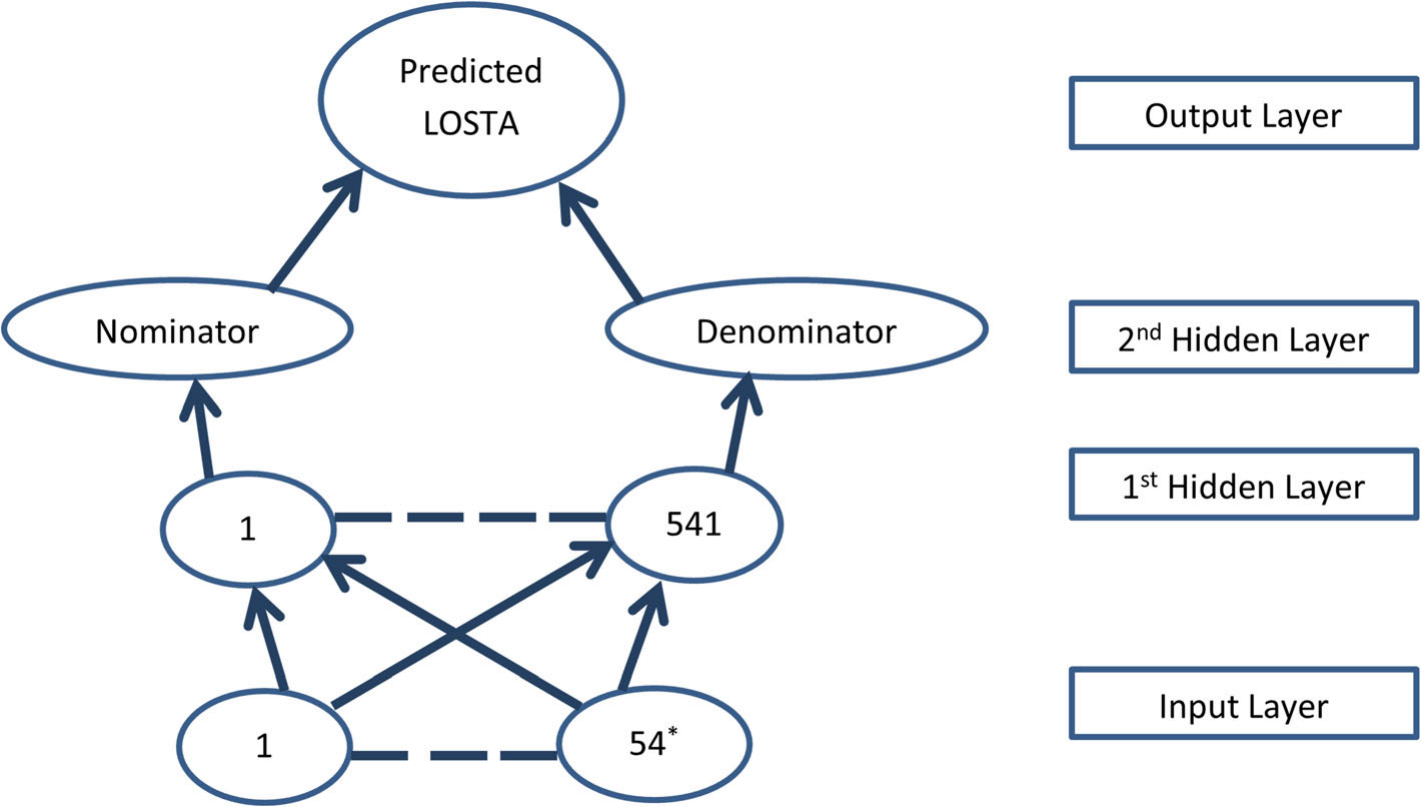
Artificial Neural Network Model Structure for the Length of Hospital Stay after the Index intervention. Endnote: ^*^number of input nodes varied as we included the significant predictors or mismatched variables predicting the outcome

We have previously described similar ANN models, their structures and the software used in another article [20]. To reiterate, in this study, Inputs included 54 variables, either matched (36 in Table 1) or unmatched (18 in Table 2), per patient. Diagnoses were made by clinicians and documented in the study form. An ANN model (Fig. 2) was developed using NeuralTools, version7.6.0 (Palisade Corp., Ithaca, NY). This was a fully connected feed forward ANN. Just like in our former studies, the first hidden layer (1 per training CPI) ensured accurate performance [20]. The second hidden layer (2 neurons, one nominator and one denominator) reduced dimensionality to drive ANN toward fast convergence (i.e., an optimal solution that can be reliably used to predict outcomes) [20]. The Input layer consisted of one neuron for each input variable. This would be 54 for the total (Fig. 2) or just 18 if only unmatched variables are included (similar to Fig. 2 with only exception of having 18 input nodes instead of 54). An additional categorical input neuron may be added for the status of CPI accepted versus rejected. Cross validation set consisted of random 4 scenarios after initial training (544, 80%) and testing (136, 20%). Therefore, data was both internally and externally cross validated. The four validation scenarios were selected at random. Sensitivity report figures showed that this distribution of training, testing and cross validation resulted in no over fitting (available upon request). Our neural tools software makes it easy to develop and analyze the ANN model. With simple icons you can select your limits for training, testing, and validation. You can also select whether your ANN is best net search, generalized regression network, or probabilistic network. Once you start the training, it will converge and stop only if one stop condition is reached. We used all possible stop conditions that is 1,000,000 training cycles, 2 min of training, or failure to further reduce the error in prediction. Reassignment and live predictions enabled the study of effect of maximizing acceptance of CPI from 80 to 100%. For example, after the results are ready you can change the inputs in the cases such that all CPI are accepted and see the effect on the length of stay. In addition, model automatically generated variable impacts (VI), an overall % contribution of a given variable to predict outcome. These were simply compared for all variables.

#### Sample size calculation

Authors used The University of California, San Francisco (UCSF) sample size calculator site (URL: http://www.sample-size.net/sample-size-study-paired-t-test/, Accessed November 27, 2019). Using Phase I data, a standard deviation of change with 80% acceptance versus 100% acceptance of CPI of 1.05 was used. At two-tailed-alpha of 5% and power 80%, a sample size of 554 consolidated CPI to detect an effect size of about 3 h (0.125 days) difference in LOSTA was needed. This effect size was based on a range from 0 days to 0.8 days seen with other research groups [14, 22]. So, in the calculator, we fed 5% for alpha (two-tailed), 20% for beta error, 0.125 days for effect size, and 1.05 for the standard error of change based on the pilot period findings. The number of CPI interventions needed would be 556 with a T statistic or 554 with a Z statistic approximation.

ANN model for the matched variables would expertly need 19*10*2 = 380 sample size. But for a larger ANN including unmatched variables the sample needed would go up to 54*10*2 = 1080. This was based on the expert approximation formula of Number of input variables included multiplied by 10 multiplied by the neurons in the second layer (i.e., 2).

#### Ethics approval

Authors obtained local ethics approval from Applied Science Private University (Approval number 2020-PHA-8). Since the data is normally collected during usual care, individual consent to participate was unnecessary. Applied Science Private University and the hospital are affiliated institutions.

## Results

### Factors associated with rejection of CPI

Critical care, pediatric age group, and non-green antibiotics were statistically matched between the two groups of accepted and rejected CPI (Table 1). We have no oncology cases and hence this specialty may not be compared between the two groups. Generally, for most mismatched variables (Table 2), there were more such variables in the rejected group. For example, stopping (42% versus 26%, *P*-value < 0.001) or adding (29% versus 21%, *P*-value = 0.027) medications were both associated with higher rejections of CPI. Clinical pharmacists with greater experience of 7 or 8 years more likely to intervened successfully (95 and 86% versus 57%). Each clinical pharmacist success rate is calculated by dividing the number of physician authorized CPI by total CPI recommended by clinical pharmacist. Respectively, these clinical pharmacists had 147, 452, and 824 successful CPI out of 256, 475, and 963 made (χ^2^ *P*-value = < 0.001). In total, our clinical pharmacists made 1423 successful out of 1694 CPI (84%). In Phase II, there were fewer rejections probably due to the clinical pharmacist with the least experience contributing fewer CPI in this phase. Generally, on a detailed analysis of every intervention made, the study panel agreed on the vast majority (> 99%) of CPI, both accepted and rejected, with the clinical pharmacist rather than the physician. This is an expert judgment rather than an assumption and has no effect on the ANN model predictions.

### ANN model convergence and LOSTA outcome

**Fig. 3:**
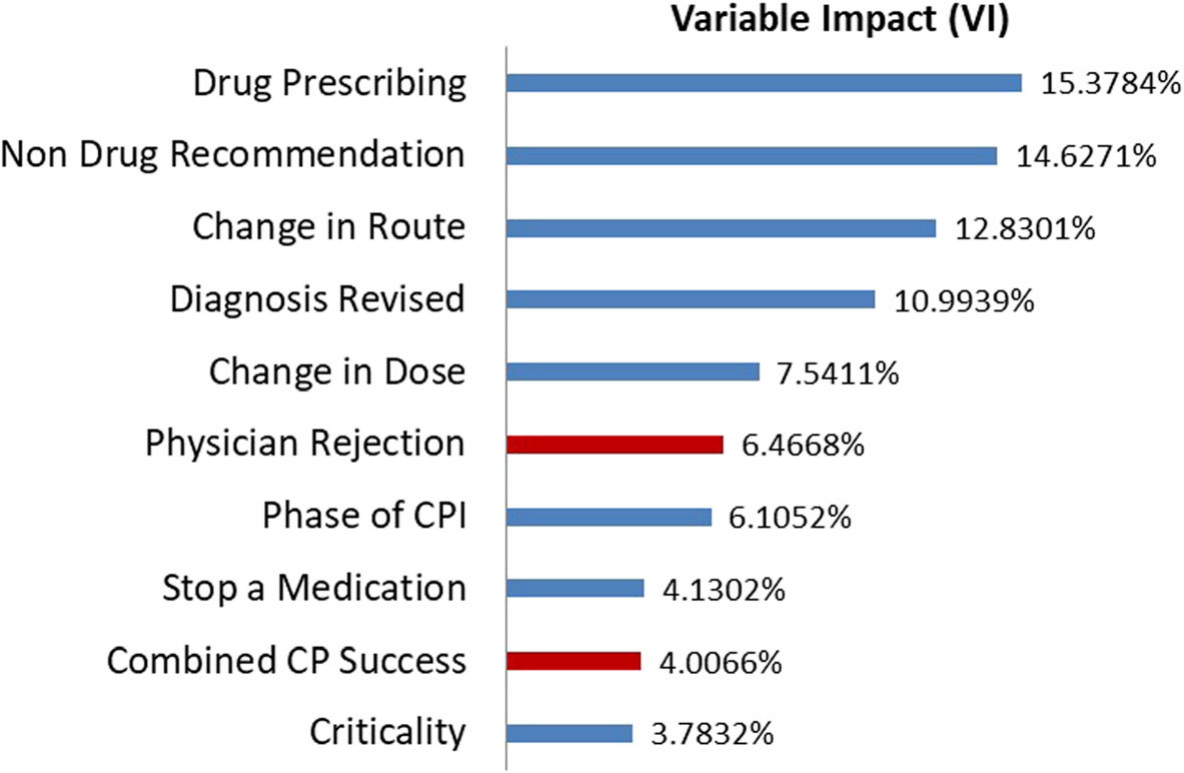
Variable Impact for Length of Hospital Stay after the Index Intervention Artificial Neural Network, all 54 input variables included

**Fig. 4:**
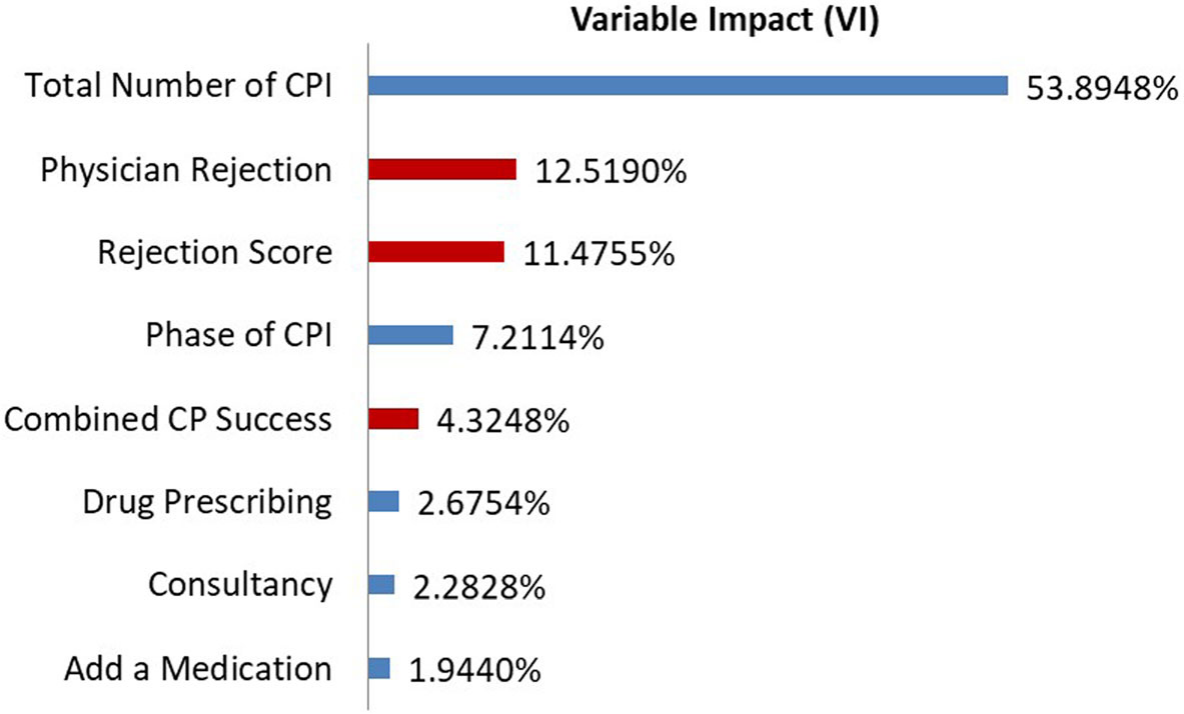
Variable Impact for Length of Hospital Stay after the index intervention, only mismatched 19 input variables included

ANN model converged within 18 s and 270 training cycles. Cross validation was very close to identity curve with accurate predictions in all models (e.g., LOSTA ANN *R*^2^ = 0.9923). Variable Impacts only for important factors are shown in Fig. 3. Rejection of CPI (combined VI of about 10%; namely 6.5% for physician rejection rate and 4% for combined CP success score) is a significant predictor of LOSTA. In this ANN model, 100% versus 80% accepted CPI were associated with 0.4 days less of hospital stay after the index CPI (2.6 ± 3.4, median (range) of 2 (0–28) versus 3.0 ± 3.8, 2 (0–30), *P*-value = 0.022). Readers may appreciate that we are giving full statistical summaries for the model as this would help assess normality of the ANN model results. Including only the 19 variables mismatched in the univariate analysis (i.e. the 18 in Table 1 plus the dichotomization binary code of accepted and rejected CPI), the ANN model converged within 3 s and 110 training cycles. The same cross validation results and LOSTA differences were observed with 100% versus 80% acceptance of CPI (2.6 ± 3.4, median (range) of 2 (0–28) versus 3.0 ± 3.8, 2 (0–30), *P*-value = 0.022). However, the number of CPI as well as the measure of their acceptance emerged as the major determinant of the LOSTA outcome. Physician rejection rate, combined CP success score, and rejection score overall explains about 28% of the variability in LOSTA (Fig. 4).

### Secondary outcomes

All secondary outcomes were insignificantly reduced with LOISCUA 0.4 ± 1.7 with median (range) of 0 (0–16) versus 0.5 ± 1.7 with 0 (0–16) (*P*-value = 0.693). Readmissions (99 out of 684) for both scenarios and 8 cases each switching to the opposite readmission category. Mortality was 1.2% versus 1.9%, *P*-value = 0.131, with only 6 and 1 cases shifting mortality status. Cost of hospitalization was reduced by a statistically insignificant average of 55 JD per consolidated CPI (2116 ± 2837 versus 2171 ± 2864, *P* value = 0.721).

## Discussion

To the best of our knowledge, this study is the first to demonstrate using an ANN model that maximizing acceptance of CPI reduces the length of hospital stay and saves hospitalization costs.

Factors that predispose a CPI to be rejected seem to vary with the setting. For example, critical care, pediatric age group, anti-infective medications, and oncology drugs are four areas with significantly higher acceptance rates of CPI in our previous governmental study [16]. In this acute care private setting, however, difference in the acceptance rates in these specialties was insignificant. Moreover, stopping a medication was more likely to be accepted in the former whereas associated with higher rates of rejections in the latter [16]. Adding a drug was more likely to be rejected in both studies. Hence, albeit unscientific, physician’s decision to accept or reject a CPI may be partially driven by practice perspective.

Somers et al. evaluated clinical pharmacy recommendations in a geriatric population [23]. They found that when physicians accepted about 54% of CPI, a panel of four physicians, pharmacologists, and clinical pharmacists accepted above 85%. Similarly, in our acute care setting, the overall acceptance rate of 84% by physicians, in fact had to be around 99% according to our researcher’s panel detailed review. These acceptance rates are consistent with other studies in the acute care setting, which were found to be in the range of 76 to 93% [24–27]. However, the clinical implications of these opposing pharmacotherapy decisions remain to be assessed. Expert judgment in this study support maximizing the acceptance of CPI but has no effect on the ANN model itself. Therefore, both our study expert panel and the results of the ANN model are concordant in anticipating better outcomes with opting to a pharmacist upper hand on prescribing upon dealing with a rejected CPI.

Redmond et al. have shown in a Cochrane meta-analysis of 20 pooled randomized clinical trials that CPI effects on secondary outcomes, including healthcare utilization, were of little certainty [28]. Similarly, our study shows that rejecting CPI has an insignificant effect on the secondary outcomes of LOSICUA and mortality. However, there was a consistent prolongation of hospitalization by about one shift or 0.4 days with these rejections. On average, the extra cost of a rejected CPI would be around 55 JD. For the study site, this would be sufficient to hire 1.27 full-time equivalents (FTE) of a clinical pharmacist. In conclusion, all three factors; namely the expert assessment, LOSTA, and cost point in the same direction in support of a 100% approval of CPI.

Limitations of this study include two important points. First, the fact that this is an initial retrospective study in this field. This point is relevant as having done a prospective study one may fail to find that higher authorization of CP interventions would reduce the length of hospital stay. Normally it would be better if we can have two groups one with 100% approval of CP recommendations (intervention group) and another matched-control group with usual 80% authorization of recommendations. Researchers can follow both prospectively to assess and measure the effect of 100% authorization of CP recommendations. Done that way, the improved authorization would relate to any observed changes in the outcomes. However, this is extremely impossible to make in our practice and would require new arrangements more than usual care. Therefore, we decided to do the study retrospectively and employ an artificial intelligence model which would give insight and possibly lay the foundation for a prospective design. Moreover, it is unlikely that the retrospective artificial intelligence design exaggerated the clinical pharmacy recommendations. Furthermore, three separate factors support our conclusion and further research in different practice settings must prospectively confirm its findings.

Second, this study proved that more authorization of CPI interventions would mean better outcomes such as reduced length of hospital stay and reduced costs of hospitalization. However, this is only one milestone in the trip to a fully independent pharmacist prescriber status. Therefore, future studies should tackle other dimensions of this privileging process such as defining the acceptable scopes of practice for individual pharmacists before blindly implementing a one-size-fit-all decision about the eligibility of pharmacists for these independent practice roles.

## Conclusion

ANN models show that maximizing acceptance of CPI reduces LOSTA and saves costs while expert judgment supports this notion in parallel. Clinicians and society will most likely benefit from maximizing the authorization of CPI. Caution should be exercised as these findings are not one-size-fit-all and may vary with setting. Finally, promoting certain competent clinical pharmacists to full independent prescriber status has many aspects to consider. Although the current study shows, in a hospital setting, that clinical pharmacists were overall well equipped to prescribe all medicines, other factors may be involved. Hence, addressing all factors such as defining the scope of practice of individual pharmacists would need to be undertaken instead of generalizations about the candidacy of the pharmacy profession itself.

## Data Availability

Data used in this study are available EXCEL spreadsheet format from the corresponding author to any researcher/scientist upon request. Data included will be those of deidentified data ready for statistics and ANN model to protect confidentiality.

## Abbreviations

ANN: Artificial Neural Networks
CP: Clinical Pharmacy
CPI: Clinical Pharmacy Interventions
ICU: Intensive Care Unit
IT: Information Technology
LOSBI: Length of hospital stay before the index clinical pharmacy intervention
LOSTA: Length of hospital stay after the index clinical pharmacy intervention
LOSICU: Length of stay in the intensive care unit
LOSICUA: Length of stay in the intensive care unit after the index clinical pharmacy intervention
LOSICUBI: Length of stay in the intensive care unit before the index clinical pharmacy intervention
VI: Variable impact (see methods under ANN model subsection for definition)
